# The overall impairment of core executive function components in patients with amnestic mild cognitive impairment: a cross-sectional study

**DOI:** 10.1186/1471-2377-12-138

**Published:** 2012-11-20

**Authors:** Dongming Zheng, Xiaoyu Dong, Hongzan Sun, Yongchuan Xu, Ying Ma, Xiaoming Wang

**Affiliations:** 1 Department of Neurology, Shengjing Hospital of China Medical University, Shenyang, 110004, China; 2 Division of Neuroradiology, Department of Radiology, Shengjing Hospital of China Medical University, Shenyang, 110004, China

**Keywords:** Amnestic mild cognitive impairment, Executive dysfunction, Neuropsychological tests, Inhibition, Working memory

## Abstract

**Background:**

It remains unclear how executive function (EF) is affected in the stage of amnestic mild cognitive impairment (aMCI). Previous studies using different methods to assess EF in patients with aMCI have reached inconsistent conclusions. The aim of the study was to explore the characteristics of EF impairments in patients with aMCI.

**Methods:**

We investigated three core components of EF (i.e., working memory, response inhibition and task switching) based on the theoretical model of EF proposed by Miyake et al. (2000) in 34 aMCI patients and 36 healthy elderly controls using computerized tasks programmed with E-prime (the 2-back task and the keep track task for working memory, the stop-signal task and the Stroop task for response inhibition and the more-odd shifting task for task switching). The overall EF and the three individual EF components were compared between groups. For EF components that were impaired, the extent of impairment was compared using a paired analysis. The aMCI group was further divided into EF-intact and EF-deficit groups according to their performances on the EF tests in clinical neuropsychological assessments. We tested for group differences among the normal controls and the EF-intact and EF-deficit aMCI groups and paid special attention to the comparisons between the EF-intact aMCI group and the control group.

**Results:**

Compared to the control group, overall EF was significantly impaired in patients with aMCI (Wilks’ λ=0.572,*P*<0.001). Four tasks (the 2-back task, the keep track task, the stop-signal task and the more-odd shifting task) that tapped the three core components of EF displayed group differences that favored the normal controls. The results of the Stroop task revealed no differences in performance between the two groups. The EF-intact aMCI patients also exhibited significantly impaired capabilities in the four tasks compared to the normal controls. There were no significant differences in the extent of impairment between the four affected tasks in the aMCI group, suggesting that the three core EF components were impaired to the same extent.

**Conclusions:**

Both the overall EF and all of the core EF components in the Miyake model of EF (working memory, response inhibition and task switching) were significantly impaired in aMCI patients, regardless of whether they had shown obvious clinical executive dysfunction.

## Background

Amnestic mild cognitive impairment (aMCI) is characterized by a decline in memory either alone or in conjunction with other domains and is a strong harbinger of eventual progression to Alzheimer's disease (AD) [1]. AD patients often exhibit various degrees of executive dysfunction [2], but it remains unclear how executive function (EF) is impaired in aMCI [3-17]. For example, Traykov et al. [15] demonstrated that task-switching and response inhibition capabilities, as evaluated by the Modified Card Sorting Test and Stroop test, were significantly decreased in MCI patients. Similarly, Perry et al. [10] reported specific problems with response inhibition and attention switching in a group of patients who were only impaired on episodic memory tests. However, in Zhang et al.’s study cognitive planning tests (Trail Making, Porteus Maze Test and verbal fluency tests) showed a group difference favoring the normal controls, but tests for inhibition (Go/NoGo task and Stroop task) failed to show a significant difference between aMCI and normal controls [16]. In contrast to all these findings, Bisiacchi et al. demonstrated preserved EF in aMCI patients compared to normal elderly people [5].

There are many reasons, such as different criteria for patient recruitment, that may account for the inconsistent conclusions of previous studies focusing on the EF of MCI. However, we believe the main reason for the diverse results is that until now, there has been no universally accepted definition of EF and, accordingly, no widely accepted standardized measure of EF. EF is not a single-component brain function, and many cognitive processes, such as response inhibition, resistance to distraction, working memory, planning, problem solving, set shifting, abstract thinking and judgment, are under the umbrella of EF [18]. There are numerous neuropsychological tests that have been declared to be measures of EF or constructs thought to be included under the EF term. However, many of them were not originally created to measure EF. For example, the Wisconsin card sort test, a frequently used clinical EF test, was originally developed to assess abstract learning and concept formation. Whether and to what extent these tests actually sample the conceptual domain of the construct of EF remains uncertain. Moreover, many EF tasks are complex and involve many cognitive processes other than EF. Thus, it is not surprising that previous studies focusing on different EF components and using different neuropsychological tests have led to inconsistent conclusions.

Given the problems mentioned above, choosing a suitable EF model is the foundation of a valid clinical study. There have been a number of EF models based on findings of factor analyses that explored the underlying dimensions of various batteries of putative EF tests to determine which combination of EF components is more representative of EF. Of these, we adopted the Miyake model (2000) [19] for the current study, in which EF is divided into three components: inhibition of prepotent responses (response inhibition), updating and monitoring of working memory representations (working memory) and shifting between tasks or mental sets (more frequently referred to as “task switching”). Using latent-variable analysis, Miyake et al. verified that the three basic executive functions were moderately correlated with one another but were distinct. Although there is controversy regarding the Miyake model, the model has been validated by studies that used different sets of EF tasks in various ages of participants [20-22] and has been widely used in the study of EF [9,23,24]. Another reason for the choice of the Miyake model is that the three component EF model is more feasible for use in clinical studies compared to models containing more components [25,26]. No previous study has examined the same three EF components of the Miyake model in aMCI patients, although there are several studies that have investigated one or two of these EF components with or without other EF components in aMCI patients [3,6,9,15-17,27]. We believe that our study, which is based on an EF model, is superior to previous studies in the selection of EF components for evaluation because in most of the previous studies, the EF components studied were determined empirically.

In this study, quantitative assessments of the three core components of EF were achieved using multiple computerized tasks. Tasks were intentionally selected to be sensitive and specific for the evaluation of a single core EF component. No other obvious cognitive processes than the target EF component were involved in the tasks. Computerized versions of the tasks had the advantage of high sensitivity compared to other versions of the same task, such as a pen-and-paper version. Based on the results of previous studies [3-12,15-17], we hypothesize that aMCI patients have significant deficits in EF. However, it is difficult to predict whether the three core components of EF are impaired universally or selectively, which is the main point of interest of this study.

## Methods

### Participants

The participants included 34 aMCI patients and 36 healthy elderly controls who were recruited through ads from the memory disorders clinic and the health examination center of Shengjing Hospital of China Medical University between June 2010 and October 2011. All participants received a detailed evaluation, including medical history, physical and neurological examinations, psychiatric and cognitive evaluations, laboratory tests, and brain magnetic resonance imaging (MRI). The Mini-Mental State Examination (MMSE) [28], the Chinese version of the Activity of Daily Living Scale (ADL) [29],the Clinical Dementia Rating scale (CDR) [30], the Montreal Cognitive Assessment (MoCA) [31] Beijing version, the Chinese version of Auditory Verbal Learning Test [32] and the 30-item Geriatric Depression Scale (GDS) [33] were used in the psychiatric and cognitive evaluations. Laboratory workups included a complete blood count and differential; serum electrolyte and glucose measures; liver and renal function tests; thyroid function tests; HIV and syphilis screening; and serum B12 and folate levels. All participants had to meet the following criteria: (1) no history or evidence of psychiatric or neurologic disease, cardiovascular disease, diabetes, thyroid disease, vitamin B12 deficiency, alcoholism or drug abuse; (2) an educational level of no less than 6 years; (3) normal or corrected-to-normal vision and normal color vision, and (4) no significant changes in conventional MRI of the brain, such as cerebral infarct, hydrocephalus or leukoaraiosis. Additionally, aMCI patients all received CDR scores of 0.5 and met Petersen’s criteria [34] for amnestic MCI, which are the following: (1) memory complaint by the patient or a reliable informant, (2) objective memory impairment as demonstrated by scores of more than 1.5 SDs below normative age and education values and (3) no global cognitive impairment and no significant impact on daily functions. For the normal controls, further inclusion criteria included a CDR score of 0 and scores in the normal range on all of the neuropsychological tests. Demographic characteristics and neuropsychological assessments of the patients and controls are presented in Table 1.

**Table 1:** Demographic characteristics and neuropsychological assessments of patients with aMCI and normal controls

	**aMCI (n=34)**	**Controls (n=36)**	***t *or χ**^**2**^	***P***	**Cohen’s *d***
Age, y	67.9 (6.7)	67.4 (5.0)	0.143	0.886	
Sex(M/Total)	14/34	18/36	0.549	0.459	
Education, y	10.0 (2.9)	11.1 (3.3)	-1.462	0.148	
MMSE	28.3 (1.5)	29.5 (0.7)	-4.279	<0.001	1.025
ADL	20.3 (0.5)	20.1 (0.3)	1.826	0.074	0.485
GDS	6.3 (1.2)	5.4 (1.1)	3.198	0.002	0.782
AVLT	15.1 (2.7)	18.5 (1.9)	-6.28	<0.001	1.456
Total immediate recall					
Long delayed recall	3.4 (0.7)	6.6 (1.4)	-12.225	<0.001	2.891
MoCA total score	20.5 (3.3)	27.3 (1.7)	-10.606	<0.001	2.595
Executive items of MoCA:					
Alternating Trail Making	0.6 (0.5)	0.9 (0.4)	-2.105	0.039	0.663
Clock Drawing Test	2.7 (0.5)	2.8 (0.4)	-1.410	0.164	0.221
Abstraction	1.0 (0.6)	1.3 (0.6)	-1.907	0.060	0.500
Verbal fluency	0.9 (0.3)	1 (0.2)	-1.436	0.157	0.392

*NC*: normal controls. Mean (standard deviation). *MMSE*: Mini-Mental State Examination.

*ADL*: Activity of Daily Living; *CDR*: Clinical Dementia Rating; *MoCA*: Montreal Cognitive Assessment; *AVLT*: Auditory Verbal Learning Test; *GDS*: Geriatric Depression Scale.

As some aMCI patients may already have executive dysfunction, the EF of aMCI patients who exhibit no obvious clinical executive dysfunction deserves special consideration. We further divided the aMCI patients into 2 groups based on their performances on the 4 EF-related tests in MoCA (Alternating Trail Making - an analogue of the Trail Making Test part B (TMT-B), the Clock Drawing Test (CDT), Abstraction and Verbal fluency) which had shown good discriminating power for EF in normal individuals [35]. The two groups were the EF-intact aMCI group (who received full marks) and the EF-deficit aMCI group (who did not receive full marks). Although the clinical evaluation of EF was relatively simple, the criterion for grouping was strict to identify aMCI patients who did not have obvious executive deficits. Finally, we included 15 patients in the EF-intact aMCI group and 19 patients in the EF-deficit aMCI group.

The study was approved by the Ethics Committee of the Shengjing Hospital of China Medical University. Written informed consent was obtained from all participants.

### EF tasks and procedures

The three core EF components were assessed by computerized tasks programmed with E-prime 2.0 (Psychology Software Tools, Inc., Pittsburg, PA). Response inhibition was assessed with a Stroop task and a stop-signal task, working memory was assessed with a 2-back task and a keep track task, and task switching was assessed with a more-odd shifting task. Responses were logged using the vocal key or buttons of the E-prime serial response box. In tasks in which both the vocal key and buttons could be used as the response modality, the vocal key was selected to maximally avoid the possible effect of the slowed motor speed of elderly participants on EF measures. Similarly, only one button of the response box was used, with simple rules for button pressing in tasks in which the vocal key could not be used. All stimuli in the experimental task were presented in size 48 font on a white background in the middle of a standard 15-in CRT computer screen. All participants were individually tested in a quiet room. They sat at a comfortable distance from the screen (generally approximately 60 cm). The order of task administration was fixed for all participants (i.e., stop-signal task, 2-back task, more-odd shifting task, Stroop task and keep track task) with the constraint that no two tasks that were intended to assess the same EF component occurred consecutively. All participants received one 5-min practice session for each task before the formal test to become familiar with requirements of the task. There was a 3-min rest period between each task. The entire test lasted approximately 1.5 h. To decrease the cognitive burden on participants, the EF assessment was not performed on the same day that the psychiatric and cognitive evaluations were performed. Typically, there are control blocks in these tasks, such as a 0-back block in the 2-back task or a block of unreadable strings of letters or symbols in the Stroop task. To shorten the total time consumed by the assessment, we did not include control blocks in this study. The lack of control blocks did not affect our study, as we were only concerned about group differences in the five performance tasks.

1. Stop-signal task: This study adopted the version of the stop-signal task used in our previous study [36]. On a Go trial, participants were instructed to press a button when they saw the Go signal (a circle). The circle disappeared when the button was pressed or after 1000 ms had passed without response, whichever came first. On a Stop trial, a Stop signal (a cross) appeared shortly after the Go signal. Subjects were instructed not to press the button on trials with a Stop signal. A Stop trial also terminated when a button was pressed or when 1000 ms had elapsed since the appearance of the Go signal. In every four trials, there were one Stop trial and three Go trials that were presented in random order. It was emphasized to subjects that quick responses to Go signals and trying to withhold responses to Stop signals were equally important. A staircase-tracking algorithm was used to modify the time interval between the Stop and Go signals (stop-signal delay, SSD) according to the responses of the participants. The SSD started at 200 ms, and if the subject succeeded in a Stop trial, the inhibition would be made more difficult on the subsequent Stop trial by increasing the SSD by 50 ms. If the subject failed to inhibit, the next Stop trial was made easier by decreasing the SSD by 50 ms. By this algorithm, approximately 50% of all Stop trials could be inhibited by subjects, which yielded accurate estimates of stop-signal reaction time (SSRT). SSRT was the dependent measure in this task. The formal test consisted of 2 blocks of 100 trials each.

2. 2-back task: In the 2-back paradigm, participants were required to monitor a series of quickly changing numbers shown on the center of the screen by pressing a button whenever a stimulus was presented that was the same as the one presented two trials previously. The number disappeared when the button was pressed or after 1000 ms had passed without response. Target numbers accounted for 25% of the stimuli, and the error rate was calculated as the dependent measure. The 2-back task consisted of two blocks of 100 trials each.

3. More-odd shifting task: In the more-odd shifting task (adapted from Salthouse et al. [37]), a series of numbers (1–4 and 6–9) were displayed on the center of the screen. Each number appeared for 1000 ms. There were two conditions in the task: (1) when the number was colored red, participants were required to say “big” as quickly as possible if the number appearing on the screen was more than 5 and “small” if it was less than 5; and (2) when the number was colored green, participants were required to say “odd” or “even” depending on the parity of the number. In the shifting block (S), which included 48 trials, participants regularly alternated between the two conditions, switching from one to the other on every two trials. Thus, the shifting block consisted of 23 switch trials and 25 non-switch trials. The control block (C) consisted of 24 trials of one condition and did not require a switch (all trials were non-switch trials). RTs were measured by vocal key, and a tape recorder was used to record the answers. Participants needed to finish two shifting blocks and four control blocks (two blocks of each condition) in the order of CCSSCC. The switch cost was the difference between the average RTs of the switch trials in the shifting blocks and the average RTs of non-switch trials in the control blocks.

4. Stroop task: In the Stroop task (adapted from Belanger et al. [3]), the Chinese characters for green, red and blue were used to construct items that were either incongruent, meaning that the name of the color differed from the ink color (e.g., the word RED written in blue), or congruent, meaning that the name of the color was similar to the ink color (e.g., the word RED written in red). Items were combined in random order to create a list in which 75% of the trials were congruent and 25% of the trials were incongruent. Participants were instructed to verbally name the color in which the word was written and to refrain from reading the printed word. They were asked to proceed as accurately and as quickly as possible. Trials terminated when participants responded or 3000 ms after the appearance of the word. RTs were measured by vocal key, and a tape recorder was used to record the answers. The main dependent measure was the RT difference between correct responses on incongruent and congruent trials. The Stroop task consisted of two blocks of 100 trials each.

5. Keep track task: The keep track task was adapted from Miyake et al. [19]. In each trial of the keep track task, participants were first shown three target categories at the bottom of the computer screen. A list of two-character Chinese words from four possible categories (i.e., animals, countries, plants and relatives) was then presented serially and in random order for 1500 ms each, and the target categories remained at the bottom of the screen. The task was to remember the last word presented in each of the target categories and then to write down these words at the end of the trial. Three trials consisting of 8, 12 or 16 words were presented twice in random order. One point was awarded for each correctly recalled word, and the total possible score was 18. The total score was the dependent measure.

### Statistical analysis

Statistical analyses were performed using SPSS 11.0. The alpha level for statistical significance was set at 0.05. T tests and χ2 tests were used to compare the demographic and clinical characteristics of the two groups. We performed a multivariate analysis of variance (MANOVA) on the five dependent measures to compare overall executive function between the aMCI patients and the controls. If the MANOVA demonstrated a significant group effect, post-hoc analyses using analysis of variance (ANOVA) were conducted to examine the between-group differences on individual EF measures. The critical α level was adjusted to 0.01 with Bonferroni correction in the post-hoc comparisons. There was a possibility that the group EF difference calculated above, if present, was caused by the decline of EF in aMCI patients that had already exhibited executive dysfunction in clinical EF tests. Therefore, all of the EF measures were compared further using ANOVA with a post-hoc Bonferroni test among the EF-intact aMCI group, the EF-deficit aMCI group and the control group. Special attention was paid to the comparison between the EF-intact aMCI group and the control group. Due to the small sample size, Cohen’s *d* was calculated to determine the effect sizes of the comparisons.

We next performed analyses to explore whether there were any significant differences in the extent of impairment on the three core executive elements in aMCI patients. To make the performances on the different tasks comparable, we transformed the performances on the different tasks into Z-scores by standardization based on the entire sample. We were interested in comparing two components. Using controls, the distribution of the difference in Z-scores can be determined for the two components. Both components may have been impaired in aMCI patients, but if the extent of impairment was the same for both component (the null hypothesis), the differences in Z-scores in aMCI patients would be similar to those in normal controls. Therefore, we performed a t-test to compare the differences in Z-scores between aMCI patients and normal controls. The added advantage of this approach was that the correlation between components that existed among some tasks (Table 2) was no longer an issue when the difference between the scores of two components was considered.

**Table 2:** Pearson correlation coefficients for the 5 measures in all participants

	**Keep track**	**2-back**	**Stop-signal**	**More-odd shifting**	**Stroop**
Keep track	-				
2-back	-0.492**	-			
Stop-signal	-0.330**	0.372**	-		
More-odd shifting	-0.422**	0.543**	0.480**	-	
Stroop	0.027	0.247*	0.028	0.238*	-

Finally, a binary logistic regression analysis was performed to assess the relative contributions of performance on each task to the classification of aMCI/controls. The healthy control group was used as the reference, and the aMCI group was used as the outcome. The five dependent measures of the above EF tasks were used as predictors.

## Results

The demographic and clinical characteristics of aMCI patients and controls are presented in Table 1. There were no significant differences in age, sex or degree of education between the patients and the controls. With regard to the clinical neuropsychological assessments, t-tests showed that the two groups significantly differed on measures of MMSE, GDS, AVLT (including total immediate recall and long delayed recall) and MoCA, which all favored the NC group (Table 1). The ADL did not differ between the two groups (*P* = 0.074, Cohen’s *d* = 0.485). With regard to the 4 EF items of MoCA, the results of the Alternating Trail Making test revealed significant group differences (*P* = 0.039, Cohen’s *d* = 0.663), but the other three tests did not (Clock Drawing Test: *P* = 0.164,Cohen’s *d* = 0.221, Abstraction: *P* = 0.060, Cohen’s *d* = 0.5, Verbal fluency: *P* = 0.157, Cohen’s *d* = 0.392).

The overall MANOVA on all task measures was significant (Wilks’ λ = 0.572,*F* = 9.572, *P*<0.001). Follow-up ANOVA revealed significant group differences on all tasks except the Stroop task. Compared to the controls, the aMCI patients exhibited poorer performance on the keep track task (*F* = 25.947, *P*< 0.001, Cohen’s *d* = 1.230), the 2-back task (*F* = 19.587, *P*< 0.001, Cohen’s *d* = 1.099), the stop-signal task (*F*= 14.513, *P*< 0.001, Cohen’s *d*= 0.910) and the more-odd shifting task (*F* = 29.502, *P*< 0.001, Cohen’s *d* = 1.297)(Table 3).

**Table 3:** Comparison of performances on the five EF tasks between aMCI and normal controls

	**aMCI (n=34)**	**NC (n=36)**	***F***	***P***	**Cohen’s *d***
Keep track	8.9 (2.6)	12.1 (2.6)	25.947	<0.001	-1.230
2-back	0.59 (0.2)	0.37 (0.2)	19.587	<0.001	1.099
Stop-signal	300.5 (31.7)	270.1 (35.0)	14.513	<0.001	0.910
More-odd shifting	353.6 (92.1)	237.9 (86.1)	29.502	<0.001	1.297
Stroop	330.7 (142.0)	319.2 (69.5)	0.188	0.666	0.103

*NC*: normal controls. Mean (standard deviation).

The ANOVA with post-hoc Bonferroni test performed among the NC, the EF-intact aMCI group and the EF-deficit group revealed that both the EF-intact aMCI group and the EF-deficit aMCI group differed significantly from the control group on all measures of EF tasks except for the Stroop task (Table 4). In the more-odd shifting task, the EF-deficit group displayed greater impairment than the EF-intact aMCI group. In the keep track task, the 2-back task and the stop-signal task, there were no differences between the two aMCI subgroups.

**Table 4:** Comparison of performances on EF tasks for EF-intact aMCI, EF-deficit aMCI and normal controls

	**NC (n=36)**	**EF-intact aMCI (n=15 )**	**EF-deficit aMCI (n= 19)**	***F*or χ**^**2**^	***P***	**NC*vs.*EF-intact aMCI**		**NC*vs.*EF-deficit aMCI**		**EF-intact-aMCI*vs*. EF-deficit aMCI**	
						***P***	**Cohen’s*d***	***P***	**Cohen’s *d***	***P***	**Cohen’s*d***
Age, y	67.4 (5.0)	68.9 (6.5)	66.5 (7.0)	0.550	0.580						
Sex(M/Total)	18/36	7/15	7/19	0.875	0.646						
Education, y	11.1 (3.3)	10.3 (2.8)	9.67 (3.0)	1.242	0.295						
Keep track	12.1 (2.6)	9.2 (2.5)	8.6 (2.6)	13.056	<0.001	0.002	1.137	<0.001	1.346	1.000	0.235
2-back	0.37 (0.2)	0.58 (0.24)	0.61 (0.22)	9.790	<0.001	0.008	0.951	0.001	1.142	1.000	0.130
Stop-signal	270.1 (35.0)	291.9 (15.4)	307.4 (39.3)	8.256	0.001	0.009	0.806	0.004	1.002	0.276	0.519
More-odd shifting	237.9 (86.1)	310.5 (29.3)	387.7 (110.0)	19.415	<0.001	0.022	1.098	<0.001	1.496	0.034	0.959
Stroop	319.2 (69.5)	332.6 (133.6)	329.2 (151.9)	0.096	0.908						

*NC*: normal controls. Mean (standard deviation). Comparison was performed using ANOVA with a post-hoc Bonferroni test among the EF-intact aMCI group, the EF-deficit aMCI group and the normal control group.

In aMCI patients, the paired analysis using Z scores did not identify significant differences in the extent of impairments between the four affected tasks, suggesting that the three core components of EF were impaired to the same extent (Table 5). The binary logistic regression analysis with the five performance measures entered as covariates demonstrated that the measures of the keep track task and the more-odd shifting task had predictive value for the identification of aMCI (OR = 1.665, with a 95% confidence interval from 1.112 to 2.494, *P* = 0.013 and OR = 0.981, with a 95% confidence interval from 0.967 to 0.995, *P* = 0.01, respectively; Table 6).

**Table 5:** Paired analysis of the differences in the performance Z-scores among the four affected tasks between the aMCI and the control groups

	***t***	***P***
Z(Keep track)- Z(2-back)	0.431	0.668
Z(Keep track)- Z(More-odd shifting)	-0.188	0.851
Z(Keep track)- Z(Stop-signal)	0.759	0.450
Z(2-back)- Z(More-odd shifting)	-0.668	0.506
Z(2-back)- Z(Stop-signal)	-0.394	0.695
Z(Stop-signal)- Z(More-odd shifting)	-1.066	0.290

**Table 6:** Summary of the Logistic regression analysis: contribution of performance in each task for the classification of aMCI/controls

	**Wald**	**OR**	**95%CI for OR**	***P***
Keep-track	6.122	1.665	1.112-2.494	0.013
2-back	0.035	0.680	0.012-37.958	0.851
Stop-signal	1.288	0.986	0.961-1.011	0.256
More-odd shifting	6.705	0.981	0.967-0.995	0.010
Stroop	1.155	1.001	0.995-1.008	0.701

*OR*: odds ratio. *CI*: confidence intervals. The control group was used as reference.

## Discussion

Although Bisiacchi et al. showed that the EF of MCI patients was intact [5], additional studies demonstrated EF deficits in patients with MCI [3,4,6-9,15]. Our study showed that overall EF of aMCI patients was significantly impaired. More importantly, this study, based on the Miyake EF model, revealed that all the three core components of EF (i.e., working memory, task switching and response inhibition) were significantly impaired in aMCI patients and that the degree of impairment in the three core components did not differ significantly. Patients who did not show executive dysfunction in clinical assessments also exhibited significantly impaired EF in computerized EF tasks that assessed the three core EF components when compared to normal controls. Recent studies have indicated that executive dysfunction is a key contributor to impairment on everyday functioning in MCI patients, even when accounting for the degree of memory deficit [11,17]. Thus, clinicians should be fully aware of the importance of making a thorough assessment of EF in aMCI patients.

The findings of this study were inconsistent with previous studies that revealed selectively impaired EF components in MCI [6,16]. As mentioned in the beginning, the inconsistency was partially caused by the evaluation of different EF components in different studies. Here, conclusions reached by the selection of different EF components were not within the scope of this discussion, and we focused only on studies that involved any of the three core EF components in the Miyake model. Although the previous studies differed greatly in the methods that were used to evaluate the two executive components, in regards to working memory and task-switching, the studies consistently showed that working memory [6,8,9,12,17,38] and task-switching [4,9,15,39-42] were impaired in MCI patients. Moreover, Aretouli et al. highlighted the role of working memory in everyday functioning in MCI patients [17]. In their study, the contribution of the three components of EF (i.e., planning/problem-solving, working memory, and judgment) to everyday functioning in patients with MCI was investigated. Only working memory contributed significantly to functional status after controlling for demographic, health-related and other cognitive factors. Ewers et al. reported that the Trail Making B test, a commonly used method to assess task switching, was one of the best predictors of conversion from MCI to Alzheimer's disease dementia [42]. Our study, using multiple computerized psychological tasks, revealed a decline in working memory and task switching in aMCI patients, including those whose EF appeared intact in clinical assessments. Meanwhile, the logistic regression analyses revealed that the performances on the keep-track task and the more-odd shifting task, which assess working memory and task switching, respectively, were potential predictors of aMCI. This conclusion will require further investigation in a large sample of patients. All of these studies, including ours, provide strong evidence of impairments in working memory and task switching in aMCI patients.

Controversy remains regarding the impairment of response inhibition in aMCI patients. Using different methods to evaluate response inhibition, some studies showed that the response inhibition of aMCI patients declined significantly compared to normal elderly controls [3,9,10,15], while other studies did not report a difference [16,27]. The results of studies that used the same task, such as the Stroop task, were inconsistent [3,15,16,27]. In our study, the results of the two tasks that evaluated response inhibition were not consistent. Performance on the Stroop task in aMCI patients was not affected, but performance on the stop-signal task was worse than in controls. In our study, the final conclusion on response inhibition of aMCI patients was based on the results of the stop-signal task, which detected a mild change in response inhibition. We believe that, in addition to the use of different criteria in patient recruitment, the main reason for the controversial results regarding response inhibition of MCI patients in previous studies and in this study is the methodological differences across these studies, such as the use of different tasks or different designs of the same task, which may differ greatly in their detection capacities. For example, in similar groups of MCI patients, Belleville et al. did not find any abnormalities in inhibition when they used the Hayling procedure and a clinical pen-and-paper Stroop task [27], while partially impaired inhibition was observed when they used a computerized Stroop task [3]. Therefore, sometimes negative results can be caused by the use of tasks with insufficient sensitivity. Selection of the most proper task or the use of multiple tasks is feasible in both clinical and research settings.

The results of our Stroop task, which was a computerized version, did not reveal the group differences that were shown by the results of the stop-signal task. The relatively simple design of our Stroop task may partially account for the insufficient sensitivity of the task. Another possible explanation is that the Stroop task itself may be less sensitive to changes in response inhibition than the stop-signal task. Response inhibition is the most extreme and straightforward form of inhibitory control and is generally required in all types of cognitive control. Our study is the first to use a stop-signal task to assess response inhibition in aMCI patients. We believe that the experimental design of the stop-signal task in our study is much better at measuring response inhibition than other inhibitory tasks, such as the Stroop task. The Stroop task is a measure of selective attention and attention control and is therefore more related to cognitive inhibition than response inhibition [43]. In a study of 112 MCI patients, Heflin et al. found that performance on the Stroop task was not correlated to disinhibition, indicating that performance on the Stroop task was a poor measurement of behavioral disinhibition [44]. In contrast, the stop-signal task rarely involves cognitive processes other than response inhibition. Meanwhile, we used a staircase-tracking algorithm in the design of our stop-signal task to ensure that approximately 50% of all stop trials could be inhibited by the subjects, which yielded accurate estimates of SSRT. This kind of quantitative evaluation of response inhibition cannot be achieved in other tasks. We speculate that it is due to these reasons that the stop-signal task detected a change in response inhibition in the aMCI patients in our study while the Stroop task did not. Decline in response inhibition can affect the capability of a patient to respond in a timely and appropriate manner to sudden changes in the external environment, such as is needed when driving.

It should be emphasized that this study was only concerned with response inhibition of aMCI patients and that other inhibitory systems, such as inhibition of thoughts and language, were not included. We cannot conclude that other inhibitory systems are similarly impaired in aMCI patients. Amieva et al. proposed that not all inhibitory mechanisms are uniformly impaired in Alzheimer’s disease. They concluded that automatic inhibition (e.g., inhibition of return) was barely affected but that controlled inhibition (e.g., stop-signal task) was greatly affected [45]. The effects of MCI and AD on different inhibitory systems need to be investigated further.

It is well known that aMCI can be classified into single domain and multiple domain subtypes depending on the profile of cognitive deficits. The so-called “single-domain” aMCI patients should have intact EF. However, as shown in some recent studies [13,14], clinically diagnosed single-domain aMCI patients exhibited significant EF deficits when they performed some special EF tasks that were not included in the routine clinical neuropsychological assessment. As this study only concerned with EF of aMCI patients and the sample size was small, we did not classify the aMCI patients into single-domain and multiple-domain subtype groups. Instead, we divided our aMCI patients into subgroups based on their performances on clinical EF tests. Compared to normal controls, not only the EF-deficit aMCI patients but also the EF-intact aMCI patients exhibited significant impairments on all the three core EF components when the more sensitive computerized tasks were used for evaluation of EF. The EF-deficit aMCI patients showed greater decreases in task switching capabilities compared to the EF-intact patients. It is highly likely that all aMCI patients have EF impairments that may differ in severity compared to normal elderly people.

It is understandable that the overall EF begins to decline in aMCI. Although atrophy of the medial temporal lobe structures is the most outstanding feature of brain atrophy in aMCI patients, recent studies on brain morphometry have shown that atrophy also exists in other brain regions, such as in the lateral and anterior parts of the temporal cortex, the posterior cingulate cortex and the frontal cortex [4,46]. There is widespread agreement that the frontal lobe, especially the prefrontal cortex (PFC), is responsible for EF. Many fMRI studies have provided strong evidence that the PFC serves as the most important neural substrate for the three core components of EF in Miyake’s model [36,47,48]. Both the atrophy of the dorsolateral PFC [49] and the impairment of white matter in the frontal lobe [50] have been shown to be related to the decline of EF in aMCI and AD patients. Therefore, pathological changes in the frontal lobe may be the basis of the decrease in all core EF components in aMCI patients.

There are some limitations to this study. First, our sample is relatively small. Second, there is a lack of follow-up data. aMCI is a heterogeneous clinical entity and not all aMCI patients will develop Alzheimer’s disease. Data from aMCI patients who progress into AD are more valuable. Follow-up work is ongoing. Third, the clinical assessment of EF is relatively simple in this study. More detailed clinical assessments of EF were not included as the present neuropsychological assessments and the five EF tasks took a long time. Thus the criteria for the selection of “EF-intact” patients are not very strict in this study. Last, the possible effect of difficulties in other cognitive functions (for instance, attention) on EF impairment was not considered in this study, but we believe it needs further thorough investigation.

## Conclusion

In conclusion, the current study showed that both the overall EF and all of the core EF components in the Miyake model of EF were significantly impaired in aMCI patients. There was no significant difference among the degrees of impairment of different core EF components. The aMCI patients who did not show obvious clinical executive dysfunction were also affected in the same way, which suggested that the impairment of EF was common in aMCI patients.

## Abbreviations

EF: Executive function; AD: Alzheimer’s disease; aMCI: Amnestic mild cognitive impairment; PFC: Prefrontal cortex; DTI: Diffusion tensor imaging; FA: Fractional anisotropy; MMSE: Mini-Mental State Examination; ADL: Activity of Daily Living; CDR: Clinical Dementia Rating; MoCA: Montreal Cognitive Assessment; AVLT: Auditory Verbal Learning Test; GDS: Geriatric Depression Scale, MANOVA: multivariate analysis of variance; ANOVA: Analysis of variance.

## Competing interests

The authors declare that they have no competing interest.

## Authors’ contributions

DZ was responsible for the conception of study, design, data analysis and the writing of the manuscript. XD, YC and YM have made contributions to data collections and analysis. HS was responsible for design and data analysis. XW was responsible for the conduction of this study. He also made contribution in conception of study and design. All authors read and approved the final manuscript.

## Pre-publication history

The pre-publication history for this paper can be accessed here:

http://www.biomedcentral.com/1471-2377/12/138/prepub
